# Lineage-specific rapid diagnostic tests can resolve *Trypanosoma cruzi* TcII/V/VI ecological and epidemiological associations in the Argentine Chaco

**DOI:** 10.1186/s13071-019-3681-7

**Published:** 2019-09-16

**Authors:** Niamh Murphy, Natalia P. Macchiaverna, M. Victoria Cardinal, Tapan Bhattacharyya, Pascal Mertens, Nicolas Zeippen, Yves Gustin, Quentin Gilleman, Ricardo E. Gürtler, Michael A. Miles

**Affiliations:** 1 Faculty of Infectious & Tropical Diseases, London School of Hygiene & Tropical Medicine, London, UK; 2 Laboratorio de Eco-Epidemiología, Facultad de Ciencias Exacta y Naturales, Universidad de Buenos Aires, Ciudad Universitaria, C1428EHA Buenos Aires, Argentina; 3 Consejo Nacional de Investigaciones Científicas y Técnicas-Universidad de Buenos Aires, Instituto de Ecología, Genética y Evolución de Buenos Aires (IEGEBA), Ciudad Universitaria, C1428EHA Buenos Aires, Argentina; 4 Coris BioConcept, Gembloux, Belgium

**Keywords:** *Trypanosoma cruzi*, ELISA, Serology, Lineage-specific, Chagas disease, Argentina, Rapid diagnostic test

## Abstract

**Background:**

*Trypanosoma cruzi*, the protozoan agent of Chagas disease, is comprised of at least 6 genetic lineages (TcI-TcVI). Their geographical distribution, clinical associations and reservoir hosts are not fully elucidated, as genotyping is hampered due to the difficulty in isolating representative populations of organisms. Lineage-specific serological techniques may address these issues.

**Methods:**

*Trypanosoma cruzi* lineage-specific serological assays were performed on human, canine, feline and armadillo sera from the Gran Chaco in northern Argentina, a region of ongoing transmission. Synthetic peptides representing lineage-specific epitopes of the trypomastigote small surface antigen (TSSA) were used in ELISA, and the TcII/V/VI shared epitope peptide (TSSApep-II/V/VI) was used in the Chagas Sero *K*-SeT rapid diagnostic test (RDT).

**Results:**

Chagas Sero *K*-SeT RDT, using Protein G to detect human and canine IgG, was at least as sensitive as TSSApep-II/V/VI ELISA using specific secondary antibodies. For sera from humans TSSApep-II/V/VI seroprevalence by Chagas Sero *K*-SeT was 273/393 (69.5%), for dogs 48/73 (65.8%) and for armadillos 1/7 (14.3%); by ELISA for cats 5/19 (26.3%). The seroprevalence for humans was similar to that for Bolivian patients, amongst whom we previously observed an association of TSSApep-II/V/VI seropositivity with severity of cardiomyopathy. In humans, prevalence of TSSApep-II/V/VI recognition was associated with locality, and with increasing and decreasing age within the Qom and Creole populations, respectively. For dogs TSSApep-II/V/VI recognition was associated with being born before community-wide insecticide spraying (*P* = 0.05) and with Qom household (*P* < 0.001).

**Conclusions:**

We show here that Chagas Sero *K*-SeT RDT can replace ELISA for TSSApep-II/V/VI serology of humans and dogs; for humans there were statistically significant associations between a positive Chagas Sero *K*-SeT RDT and being resident in Area IV, and for dogs association with Qom household or with being born before the mass spraying campaign; we also show that with cats the TcII/V/VI epitope can be detected by ELISA. We assessed the lineage distribution in an unprecedented 83% of the human *T. cruzi*-seropositive population. These results form the basis for more detailed studies, enabling rapid in-the-field surveillance of the distribution and clustering of these lineages among humans and mammalian reservoirs of *T. cruzi* infection.

## Background

Chagas disease, caused by infection with the protozoan parasite *Trypanosoma cruzi*, remains a major public health problem in endemic regions of Latin America. The initial acute phase of infection may be asymptomatic or have mild and non-specific symptoms but can be fatal, particularly in infants, young adults or the immunocompromised. Without successful treatment *T. cruzi* infection is life-long: the immune response reduces the level of infection but is unable to eliminate it, as is apparent from xenodiagnosis or PCR of seropositive patients, and recrudescent parasitaemia in the immunocompromised. In the chronic phase, around 30% of those infected will develop chagasic heart disease, and a proportion will also have gastrointestinal megasyndromes [1, 2]. Of the 1.5 million people in Argentina infected with *T. cruzi*, approximately 370,000 are estimated to have chagasic cardiomyopathy [3].

Ongoing transmission is primarily maintained by contamination with *T. cruzi* infected faeces of the predominant local triatomine insect vector, *Triatoma infestans*, which infests rural dwellings, especially in the Gran Chaco region, where vector control has had limited success [4]. Transmission can also be oral by consumption of triatomine faeces-contaminated food or congenitally, and *via T. cruzi* infected blood or organ donors. *Trypanosoma cruzi* infection is a zoonosis: dogs, cats and rodents associated with households are reservoir hosts, with evidence of a positive association between the number of infected dogs and the prevalence of human infection [5]. A wide range of sylvatic mammals carry *T. cruzi* infection [6].

*Trypanosoma cruzi* is currently understood to comprise six genetic lineages TcI-TcVI [7], with TcBat proposed as a seventh lineage, related to TcI [8]. Based on genotyping, TcII/V/VI lineages predominate in the domestic cycle in southern cone countries, including Argentina. However, genotyping may be biased by non-representative isolation of *T. cruz*i, which has sequestered intracellular replication and only scanty chronic blood infections, and by competitive selection *in vitro* between the lineages.

The polymorphic trypomastigote small surface antigen (TSSA), expressed on bloodstream trypomastigotes, has been the only antigen applicable for indirect, serological identification of lineage(s) carried by a patient or reservoir host [9]. TcI, TcIII and TcIV each have their own distinct potential TSSA epitope. At the same site a distinct amino acid sequence is shared by TcII/V/VI, and the hybrids TcV/VI also have a second sequence, as they are heterozygous and have two haplotypes at that locus [10]. Recombinant TSSA produced in *E. coli* or synthetic peptide epitopes (TSSApep) have been used with Argentine chagasic samples for *T. cruzi* lineage-specific serology [9, 11–21], particularly with the isoform common to TcII/V/VI; the recombinant form has also been used for canine serology [12, 22].

We recently developed the novel rapid diagnostic test (RDT) Chagas Sero *K*-SeT incorporating TSSApep-II/V/VI and found that response to this RDT was associated with severity of cardiomyopathy in Bolivian patients [23]. As Chagas Sero *K*-SeT uses Protein G to detect IgG, this same test should be directly applicable to both humans and diverse mammal species.

Here, our objectives were to apply *T. cruzi* lineage-specific TSSApep ELISA and the Chagas Sero *K*-SeT RDT to humans and mammals of the Chaco region of northern Argentina to gain further insight on ecological and epidemiological associations, focusing here on TcII/V/VI.

## Methods

Serum samples from seropositive patients and *T. cruzi*-infected animals were from archives stored at the University of Buenos Aires.

### Study sites

The two study sites were the municipalities of Pampa del Indio and Avia Terai in Chaco Province, northern Argentina. The majority of samples tested were from a larger ongoing project on the eco-epidemiology and control of Chagas disease, taking place in the rural area of Pampa del Indio (1600 km^2^), consisting of 1446 inhabited households in 30 villages [24]. There are two main ethnic groups inhabiting the area, Creole and Qom; the latter make up half of the local population, but are unevenly distributed among the rural villages [25]. For logistic reasons we divided the rural area into 4 study areas (named Areas I-IV). Vector control activities included a baseline house infestation assessment, followed by a community-wide spraying with pyrethroid insecticides, which took place between 2007–2009, complemented by periodic entomological surveys and community-based surveillance to detect re-infestation [5, 26–28].

**Fig. 1:**
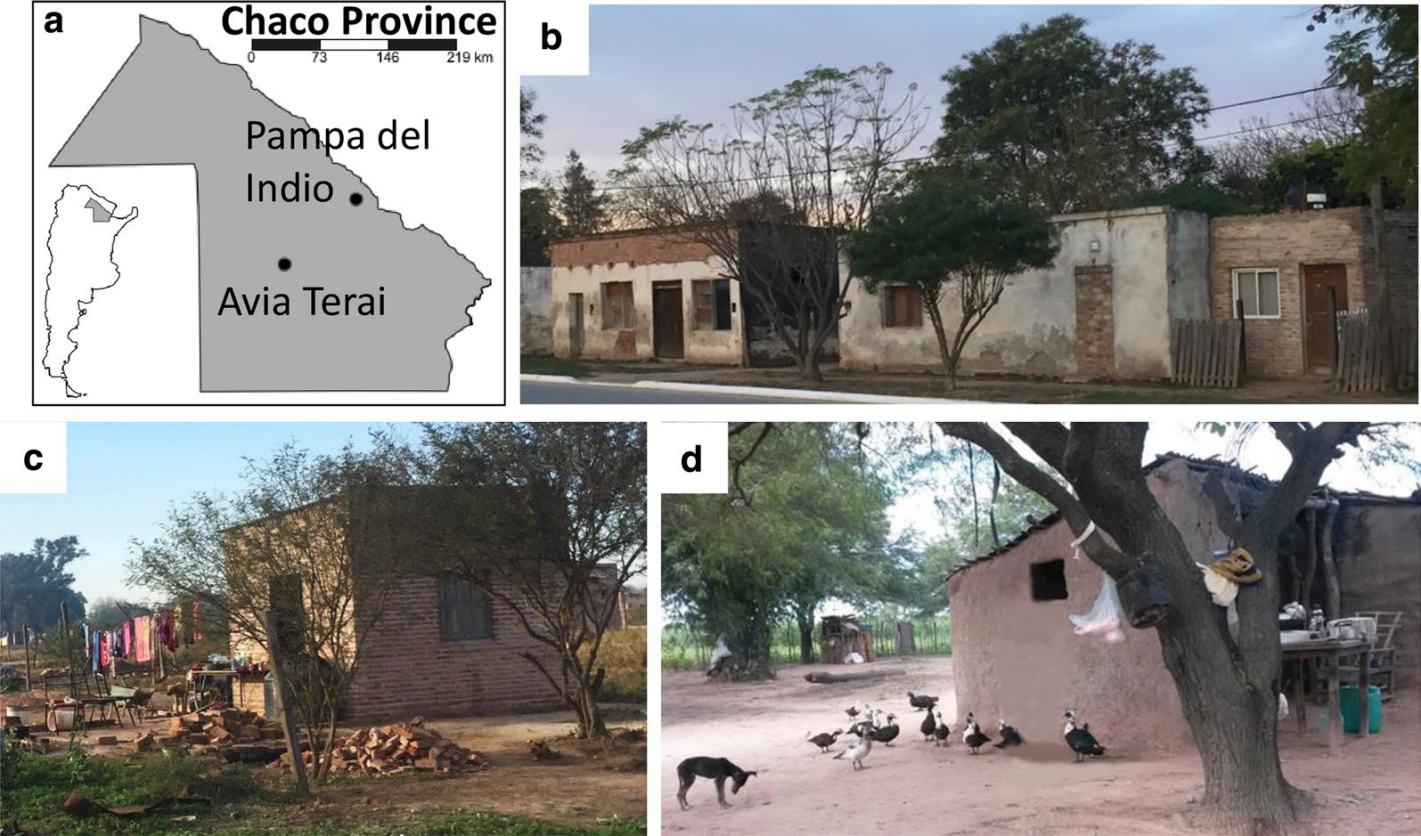
Study setting. **a** Location of Pampa del Indio and Avia Terai study sites in Chaco Province, Argentina. **b** Typical periurban dwelling. **c**, **d** Typical rural dwelling and environment in Avia Terai (**c**) and Pampa del Indio (**d**)

In 2015, research activities were expanded to include Avia Terai municipality (770 km^2^), around 150 km from Pampa del Indio. This municipality comprises 307 rural households, inhabited by a Creole population. Figure 1 shows typical dwellings and environment of the study sites.

### Sample collection

#### Human samples

*Trypanosoma cruzi* seropositive human samples were obtained in different serosurveys that took place from August 2014 until July 2017. Serum samples were examined using conventional serology by means of two ELISAs using either semipurified fractions of epimastigote lysate (Chagatest, Wiener lab, Argentina) or recombinant antigens (ELISA Rec V3.0, Wiener lab). A patient was considered Chagas seropositive if reactive in both tests. Serologically discordant samples were tested by an indirect immunofluorescence antibody test (IFAT) (Ififluor Parasitest Chagas, Laboratorio IFI, Buenos Aires, Argentina) or submitted to the reference diagnosis laboratory at the National Institute of Parasitology “Dr. Mario Fatala Chabén” (Buenos Aires, Argentina) for a final diagnosis. In addition, 10 *T. cruzi* seronegative human samples from Buenos Aires (a non-endemic area) presenting with other pathologies and 20 seronegative samples from the study sites were assayed by Chagas Sero *K*-SeT.

#### Animal samples

In 2008, cross sectional house-to-house surveys were carried out targeting all dogs and cats within 7 contiguous villages of Pampa del Indio considered to have a high infestation of *T. cruzi* infected triatomine bugs. Owners were interviewed *via* questionnaire and asked for further information on whether they had permanent residence in the study village or came from other villages outside the study area [25]. Additional samples were collected during a dog survey carried out in June 2016 (Cardinal et al., unpublished). Dogs and cats ≥ 4 months of age were examined by serology and younger animals and cats were examined by xenodiagnosis. Up to 7 ml of blood were taken from the animals by trained and experienced field personnel, and processed and stored as previously described [29]. A dog or cat was considered infected with *T. cruzi* if it was seroreactive with at least two serological tests (i.e. seropositive by ELISA and indirect haemagglutination test) or if it was xenodiagnosis-positive.

*Trypanosoma cruzi*-infected armadillos were captured using traps baited with beef or chicken strips soaked in fish sauce in different trapping surveys from August 2008 to August 2011. Traps were checked every morning and re-baited when needed. Full capture and sampling methods are described elsewhere [30]. Armadillos were examined for infection by xenodiagnosis as described [31] and not by conventional serology.

### TSSA lineage-specific serology

A total of 393 human, 85 dog (*Canis familiaris*), 19 cat (*Felis catus*) and 7 armadillo (6 *Dasypus novemcinctus* and 1 *Tolypeutes malacus*) serum samples were tested here by TSSApep lineage-specific ELISA and/or the Chagas Sero *K*-SeT RDT. A subset of 38/393 human and 73/85 dog serum samples were tested by both TSSApep-II/V/VI ELISA and Chagas Sero *K*-SeT. All these human and dog samples tested by both lineage-specific serology methods were positive by conventional serology.

### TSSApep lineage-specific ELISA

ELISAs were performed with synthetic peptides TSSApep-II/V/VI, -III, -IV and -V/VI representing residues 37–52 in the TSSA protein of those lineages (Additional file 1: Table S1) and with a control reference *T. cruzi* TcII lysate (IINF/PY/00/Chaco23) as described previously [17], with the modifications described below for human, canine and feline samples. In all cases, two replica plates were run simultaneously. Cut-offs were determined by first subtracting the plate background (no antigen wells) absorbance values from the mean reading for each sample; those samples that were then greater than five standard deviations higher than seronegative controls were considered positive.

#### Human samples

This was performed as described previously [17], with the following modifications: 0.1 μg of each TSSApep was used per well; goat anti-human IgG-HRP (074-1006: SeraCare, USA) diluted 1:5000 was used; reaction wells were developed with 100 μl of ABTS substrate (50-62-00: SeraCare) and stopped with 50 µl of stop solution; absorbance values were determined at a wavelength of 405 nm.

#### Dog and cat samples

ELISA plates were coated directly with each TSSApep at 0.1 μg/100 μl / well in coating buffer overnight. After blocking and washing steps as described [17], 100 μl of 1:200 (dog) or 1:500 (cat) dilutions of sera were applied. Subsequently, 100 μl of goat anti-dog IgG-HRP (14-19-06, SeraCare) diluted 1:12,000, or goat anti-cat IgG-HRP (14-20-06, SeraCare) diluted 1:5000, was used, prior to addition of substrate.

### Chagas Sero *K*-SeT RDT

This novel RDT, manufactured at Coris BioConcept, employed TSSApep-II/V/VI as the antigen and Protein G as the detection molecule for IgG, as previously described [23]. Tests were visually assessed at 15 min maximum incubation time and considered valid if the control band was present; the additional presence of a test line band of any intensity was considered positive for TSSApep-II/V/VI recognition. The absence of test line band was considered a negative test. The presence of the test band was determined by visual inspection of the RDT, independently by two individuals.

### *Trypanosoma cruzi* genotyping

*Trypanosoma cruzi* lineage was determined by PCR of the genomic targets spliced-leader (SL) DNA, 24Sα ribosomal RNA genes and A10 from *T. cruzi* isolates [32, 33]. For humans only, a second PCR-based protocol targeting two nuclear genes (TcSC5D and TcMK) [34] was also employed [35] to allow for classification of lineages TcI-TcVI as well as TcBat and TcV/VI [34].

### Statistical analyses

Fisher’s exact test (two tailed) was used to calculate odds ratios, 95% confidence intervals and *P-*values (StataCorp. 2019. Stata Statistical Software: Release 15. StataCorp LLC, Texas, USA). A *P-*value ≤ 0.05 was considered significant. A Kappa test was used to determine the level of agreement between the TSSApep-II/V/VI ELISAs and Chagas Sero *K*-SeT RDT, the degree of agreement was qualified by Kappa and categorized as mild, moderate or severe and 95% confidence intervals calculated (GraphPad, San Digeo, USA). For seropositive humans from Area II and IV, we performed univariate and multivariate (generalized linear model, GLMs) analyses to detect factors associated with RDT reactivity by means of a logistic regression. The full model tested was: RDT reactivity ~ age at diagnosis *vs* ethnic group + study area + gender + occurrence of *T. infestans* in the household + another cohabitant with reactive RDT. Linear regressions were calculated for each ethnic group. For Creoles, we forced the origin in 100%. Univariate analysis of dog RDT reactivity was performed for animals examined for diagnosis in 2008.

## Results

A total of 373 human, 85 dog and 19 cat samples were seropositive as described in Methods. Seven armadillos were positive by xenodiagnosis. Additionally, 20 human samples were seronegative by conventional serology. Most (292/393, 74.3%) of the human samples belonged to 10 rural villages in Area II and Area IV from Pampa del Indio, where we aimed at full coverage of the detected seropositive population. In these villages a total of 1338 inhabitants were serodiagnosed and 332 (24.8%) found seropositive for *T. cruzi* (Macchiaverna et al., unpublished) with 88.0% (292/332) of these seropositive patients assayed by Chagas Sero *K*-SeT RDT.

### Chagas Sero *K*-SeT is more sensitive than TSSApep-II/V/VI ELISA for humans and dogs

**Fig. 2:**
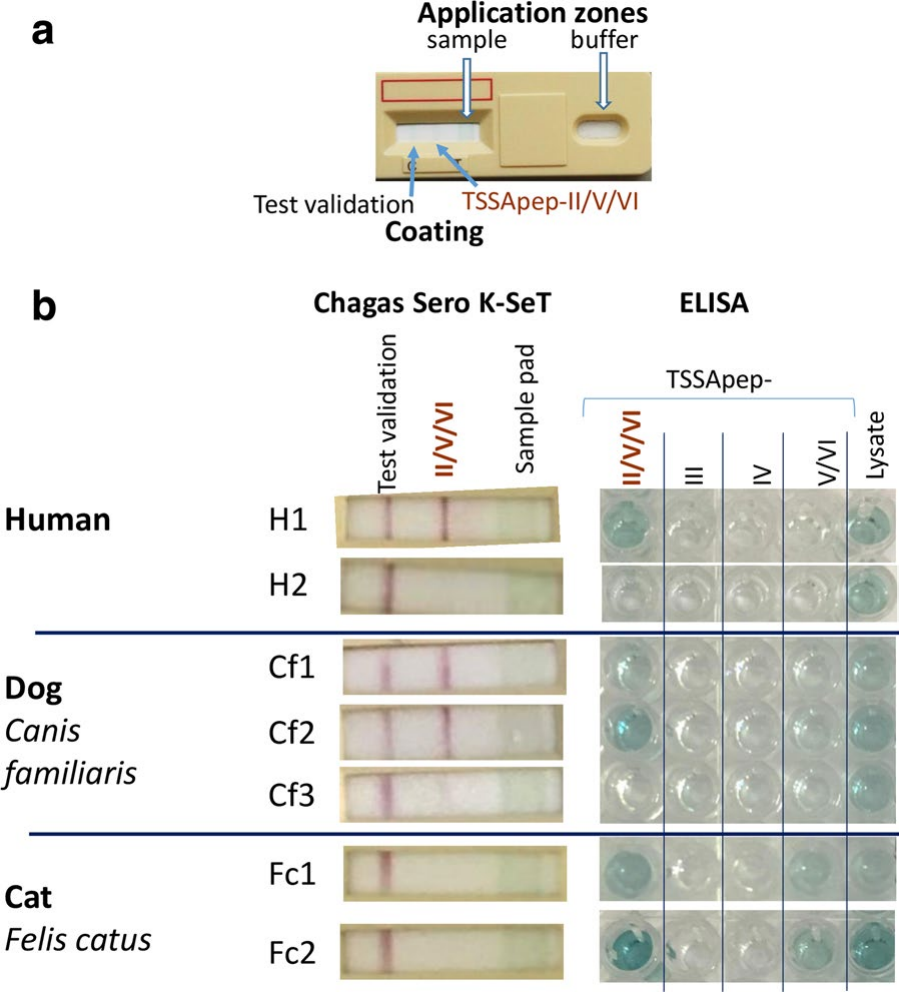
Comparison of Chagas Sero *K*-SeT RDT and ELISA for TSSApep-II/V/VI serology. **a** Chagas Sero K-SeT RDT, which uses Protein G for IgG detection, showing coating of nitrocellulose membrane and zones for application of sample then buffer. **b** Results were assessed visually and accorded with ELISA using specific secondary antibodies of both humans and dogs (samples H1 and Cf1, Cf2 positive by both tests; H2 negative by both tests), and was in some cases more sensitive than ELISA (sample Cf3: positive RDT, negative ELISA). Some dog and cat sera were positive by ELISA for TSSApep-V/VI in addition to TSSApep-II/V/VI (samples Fc1 and Fc2). Chagas Sero *K*-SeT was not able to detect feline IgG (samples Fc1, Fc2: positive ELISA, negative RDT). ELISA results were based on absorbance unit values and the cut off was determined by comparing to *T. cruzi* seronegative samples

Comparing the TSSApep-II/V/VI ELISA and the Chagas Sero *K*-SeT RDT, all human samples that were positive by TSSApep-II/V/VI ELISA were also positive by Chagas Sero *K*-SeT RDT for recognition of this peptide; Figure 2 shows examples of correspondence between these methods. However, this RDT additionally identified 10 human samples as positive that were negative by TSSApep-II/V/VI ELISA (Table 1), although seropositive by conventional serology. Thus, for human samples tested by both methods, 13/38 (34%) were TSSApep-II/V/VI ELISA positive whereas 23/38 (61%) were positive by Chagas Sero *K*-SeT. Consequently the Kappa statistic showed moderate agreement between the two tests (0.51; 95% CI: 0.28–0.74). Similarly, all dog samples that were positive by TSSApep-II/V/VI ELISA were also positive by Chagas Sero *K*-SeT RDT (Fig. 2); among these samples tested by both methods, 33/73 (45%) were TSSApep-II/V/VI ELISA positive whereas an additional 15 were positive by Chagas Sero *K*-SeT only (48/73; 66%). Here, the Kappa statistic found a good agreement between the two tests (0.60; 95% CI: 0.44–0.77). Furthermore, the Protein G conjugate in Chagas Sero *K*-SeT was highly effective in detecting binding of both human and canine IgG to TSSApep-II/V/VI, without the need for the specific secondary antibodies used in the ELISA.

**Table 1:** Human, dog and cat samples assayed by TSSApep ELISA and/or Chagas Sero *K*-SeT RDT

Host	Assayed by both ELISA and RDT				Individual assay positives				
	RDT and ELISA positive	RDT only positive	ELISA only positive	RDT and ELISA negative	II/V/VI ELISA	III ELISA	IV ELISA	V/VI ELISA	RDT
Human	13/38	10/38	0	15/38	13/38	0	0	0	273/393 (69.5%)
Dog	33/73	15/73	0	25/73	33/85	0	0	12/85^a^	48/73 (65.8%)
Cat	0	0	2/2	0	5/19	0	0	5/19^a^	0/2

^a^Also positive by TSSApep-II/V/VI ELISA

### TSSApep ELISA

Table 1 shows the TSSApep lineage-specific ELISA results for human, dog and cat samples. For humans, ELISA reaction to TSSApep-II/V/VI occurred with 13/38 (34.2%) sera, whereas it was 33/85 (38.8%) for dog and 5/19 (26.3%) for cat. Interestingly, of these animal samples 12 dog and all 5 cat samples were additionally positive for TSSApep-V/VI, which differs from TSSApep-II/V/VI by a single amino acid substitution (Additional file 1: Table S1).

### Chagas Sero *K*-SeT RDT

In total across the two study sites the detected prevalences of TcII/V/VI infection by Chagas Sero *K*-SeT in humans (273/393, 69.5%) and dogs (48/73, 65.8%) were similar (Table 1). However, as expected due to the lack of Protein G efficacy with cats, Chagas Sero *K*-SeT was negative with sera of 2/2 cats strongly seropositive for TSSApep-II/V/VI by ELISA (Fig. 2). For armadillos, 1/7 (14.3%) was weakly positive with Chagas Sero *K*-SeT (Fig. 2).

### Comparison of lineage-specific serology with genotyping

Corresponding *T. cruzi* genotyping data were available for a subset of the human, dog, cat and armadillo samples tested by TSSApep lineage-specific serology (Additional file 1: Table S2). A total of 28 of 38 human serum samples with genotyping data were reactive by Chagas Sero *K*-SeT. Given that all human-infecting lineages were genotyped as TcV or TcVI, the sensitivity of Chagas Sero *K*-SeT was 73.7% (95% CI: 57.8–85.1%). None of the 10 *T. cruzi* seronegative serum samples from non-endemic patients with other pathologies was reactive by Chagas Sero *K*-SeT. However, 8 of 20 sera from the study sites that were negative with our conventional serology were reactive by Chagas Sero *K*-SeT. Overall, 8/30 samples Chagas seronegative by conventional serology were reactive by the RDT, thus estimated specificity was 73.3% (95% CI: 55.5–86.0%).

Of the 17 dogs for which the *T. cruzi* genotype was TcII/V/VI or TcVI, 11/17 were positive for TSSApep-II/V/VI by Chagas Sero *K*-SeT (7 were TSSApep-II/V/VI ELISA positive only, 4 were additionally TSSApep-V/VI positive, and 6 were TSSApep ELISA negative); 2/17 were negative by both lineage-specific serological methods. For the single dog from which TcIII was genotyped, the corresponding serum was Chagas Sero *K*-SeT positive but TSSApep ELISA negative.

Of the four cats for which *T. cruzi* was genotyped as TcII/V/VI or TcVI, all were negative by TSSApep ELISA. The single armadillo that was TSSApep-II/V/VI positive by Chagas Sero *K*-SeT had *T. cruzi* genotyped as TcIII, as were the remaining armadillos for which these genotyping data were available.

### Hosts, clustering and ecological associations

Among the two sites (Pampa del Indio and Avia Terai), in Pampa del Indio 242/350 (69.1%) were positive by Chagas Sero *K*-SeT compared to 31/43 (72.1%) in Avia Terai, but this was not statistically significant (OR: 0.8; 95% CI: 0.4–1.7; *P* = 0.69).

Univariate associations of TSSA-II/V/VI seropositivity by Chagas Sero *K*-SeT within the Pampa del Indio study Areas II and IV (humans and dogs) are shown in Table 2. For humans, there were no significant associations between TSSApep-II/V/VI recognition and age, ethnicity, previously infested house, gender or having another householder TSSA-II/V/VI positive. A significantly higher Chagas Sero *K*-SeT reactivity was observed for patients inhabiting Area IV compared to Area II (OR: 2.07; 95% CI: 1.15–3.88; *P* = 0.02).

**Table 2:** Univariate analyses of hosts, clustering and ecological associations with Chagas Sero *K*-SeT (Pampa del Indio)

	Category	*n*	No. positive (%)	OR (95% CI)	*P-*value
Humans	Age	292	199 (68.2)	1.00 (0.98–1.01)	0.62
	Ethnicity				
	Creole	68	50 (73.5)	1	
	Qom	224	149 (66.5)	0.72 (0.38–1.29)	0.28
	Gender				
	Female	144	100 (69.4)	1	
	Male	148	99 (66.9)	0.89 (0.54–1.46)	0.64
	Study area				
	II	212	136 (64.2)	1	
	IV	80	63 (78.8)	2.07 (1.15–3.88)	0.02*
	Presence of *T. infestans* in the household				
	No	59	43 (72.9)	1	
	Yes	233	156 (67)	0.75 (0.39–1.40)	0.38
	Cohabitant with reactive RDT				
	No	146	102 (69.9)	1	
	Yes	146	97 (66.4)	0.85 (0.52–1.40)	0.53
Dogs	Function				
	Guardian	41	26 (63.4)	1	
	Hunting	32	23 (71.9)	1.47 (0.49–4.59)	0.47
	Type of hunting				
	Sylvatic animals	34	23 (67.6)	1	
	Not hunting	16	11 (68.8)	1.05 (0.25–4.84)	1.00
	Place of birth				
	In study area	52	36 (69.2)	1	
	Not in study area	12	9 (75)	1.33 (0.28–8.63)	1.00
	Ethnicity of the household				
	Creole	57	31 (54.4)	1	
	Qom	22	20 (90.1)	8.39 (1.73–78.92)	0.00*
	Born after mass spraying				
	Yes	5	1 (20)	1	
	No	73	50 (68.5)	8.70 (0.78–436.49)	0.05*
	Place of sleeping				
	Outside of the domicile	18	12 (66.7)	1	
	Inside of the domicile	14	12 (85.7)	3.00 (0.41–35.07)	0.41

*Note*: Areas II and IV only. In each case, only those samples where the examined information was known were included in the analysis

* Statistical significance (*P* ≤ 0.05)

*Abbreviations*: OR, odds ratio; CI, confidence interval

For dogs born before the mass insecticide spraying programme there was a significant association with TSSApep-II/V/VI seropositivity (OR: 8.70; 95% CI: 0.78–436.49; *P* = 0.046). Interestingly, unlike for humans, Qom household was also significantly associated with TSSApep-II/V/VI recognition in dogs (OR: 8.39; 95% CI: 1.73–78.91; *P* = 0.003). We found no evidence of significant associations between TSSApep-II/V/VI recognition and the roles and behaviour of dogs, such as sleeping inside or hunting (Table 2).

For the 19 cats that were assessed according to the available information, there were no significant associations between recognition of TSSApep-II/V/VI or TSSApep-V/VI, Qom or Creole ownership, hunting, domestication and sleeping habits (data not shown).

**Fig. 3:**
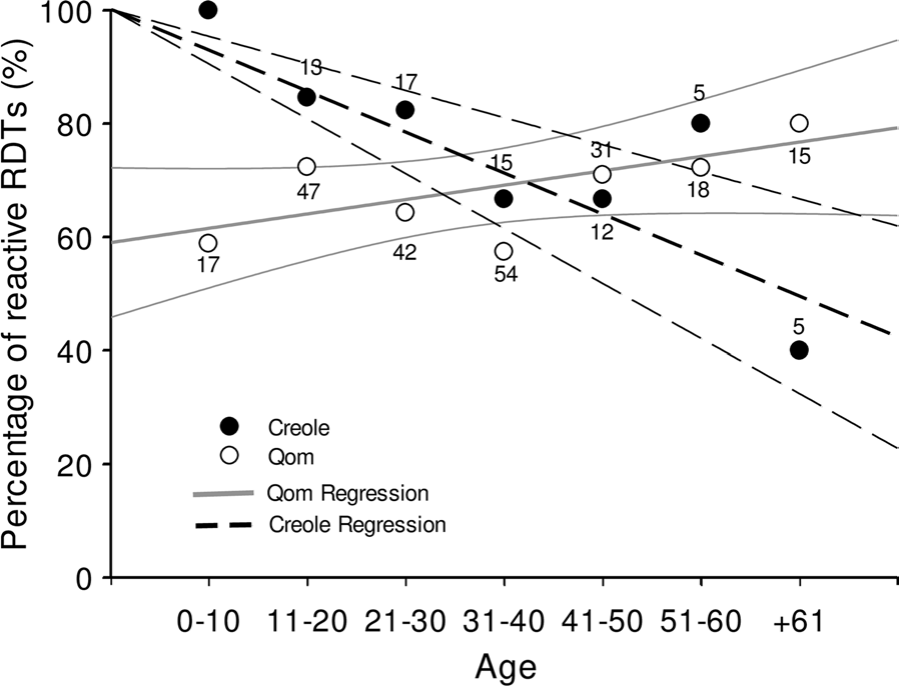
Chagas Sero K-Set seroreactivity by age and ethnicity, Areas II and IV, Pampa del Indio. Numbers above dots indicate serum samples analysed for each category. Lines represent linear regressions for each ethnicity

By means of multivariate regression analysis variables associated with Chagas Sero *K*-SeT seropositivity were identified. A significant interaction between age and ethnicity was observed: for Qom the reactivity increased with age, whilst for Creoles it decreased (Table 3 and Fig. 3). For Creoles, the percentage of RDT reactive persons decreased with age with a significant slope of − 0.72 * age (in years) (*R*^2^ = 70.34, *P* = 0.0003) whereas for Qom the reactivity increased with a slope of 0.25*age, though it was marginally significant (*R*^2^ = 45.27, *P* = 0.098). As observed in the univariate analysis, inhabitants from Area IV exhibited a higher reactivity than those from Area II. No significant associations were observed with the other variables evaluated (Table 3).

**Table 3:** Multivariate analyses for associations with Chagas Sero *K*-SeT, Areas II and IV, Pampa del Indio

Category	OR (95% CI)	*P-*value	RI
Age	0.98 (0.93–1.02)	0.31	0.48
Ethnicity			0.45
Creole	1		
Qom	0.38 (0.05–3.06)	0.36	
Gender			0.27
Female	1		
Male	0.93 (0.56–1.55)	0.78	
Area			0.82*
II	1		
IV	2.05 (1.08–3.88)	0.03*	
Presence of *T. infestans* in the dwelling			0.41
No	1		
Yes	0.69 (0.36–1.32)	0.26	
Cohabitant with reactive RDT			0.32
No	1		
Yes	0.82 (0.49–1.38)	0.45	
Age *vs* ethnicity			0.23
Age *vs* Creole	1		
Age *vs* Qom	1.04 (1.00–1.09)	0.04*	

*Abbreviations*: OR, odds ratio; CI, confidence interval; RI, relative importance

* Statistically significant

## Discussion

We have previously applied TSSApep lineage-specific ELISA to human chagasic sera [17] and to sylvatic primate hosts of *T. cruzi* [36], and adapted TSSApep-II/V/VI serology to the Chagas Sero *K*-SeT RDT [23]. Here, we deployed TSSApep serology as a rapid and efficient means for surveillance of *T. cruzi* lineage distribution among humans and animals in active transmission cycles in the Chaco region of northern Argentina.

Previous reports using TSSA serology on Argentine chagasic samples have been principally based on ELISAs and immunoblotting [9, 11–16, 18–21]. Here, we applied TSSApep-II/V/VI serology in a user-friendly, low cost RDT format, applicable at point-of-care to patients. We show excellent concordance between the performance of the Chagas Sero *K*-SeT and TSSApep-II/V/VI ELISAs in humans, as also seen with Bolivian sera [23]. However, more samples tested by both lineage-specific methods were positive with the RDT, suggesting potentially either a greater sensitivity or lower specificity. However, *in silico* analysis and sequencing of the TSSA gene from *T. cruzi* encompassing a range of hosts and geographical locations has not identified any novel epitopes (unpublished observations). Furthermore, none of the 30 seronegative samples was positive by this RDT, indicating that the RDT has greater sensitivity, as might be expected because the RDTs employ higher serum concentrations. Using the observed sensitivity and specificity we estimated that the prevalence of infection with TcII/V/VI in the seropositive human population of Pampa del Indio is 88.2% (95% CI: 76.4–99.0%), which provides further support for the prevalence of hybrid lineages in infected humans from the Chaco, as indicated by time-consuming artificial xenodiagnosis, *in vitro* culture, parasite isolation and PCR-based lineage identification [35].

Interestingly, the prevalence of Chagas Sero *K*-SeT RDT positives in these Argentine patients (69.5% for Pampa de lndio and Avia Terai combined) is similar to that seen in Bolivian patients (66.9%) amongst whom we observed an association with severity of cardiomyopathy [23]. Moreover, the significant differences observed in the Chagas Sero *K*-SeT RDT reactivity between study Areas II and IV of Pampa del Indio and the different trends for age in the two ethnic groups merits further study, given that this may be related to different incidence rates of cardiomyopathy.

One strength of this study is the level of coverage of the seropositive human population achieved. Overall, considering Area II and IV from Pampa del Indio, 59.9% of seropositive inhabitants yielded *T. cruzi* lineage identification. There is no precedent in the literature of such coverage in a well-defined human population. Most of the previous lineage identification studies comprised human samples collected in hospitals; therefore, the geographical or epidemiological context where the infection originated remains unclear [37–44]. Another constraint for mass lineage identification is the complexity of the traditional genotyping methods, which usually require *T. cruzi* isolation or large blood samples.

There was no association between ethnic group and overall human seroprevalence in Areas II and IV of Pampa del Indio. Nevertheless, Qom communities in Area III are predicted to have higher seroprevalence than Creole communities because of their lower formal education level, tendency not to apply insecticides and lack of screened windows [45]. Creole households have been observed to have a substantially lower risk of triatomine bug and dog infection compared to Qom households in Area I [25].

We also demonstrate that Chagas Sero *K*-SeT is applicable, without modification, to dogs. In two previous studies on *T. cruzi* lineage-specific serology in Argentine dogs [12, 22], recombinant TSSA-II/V/VI protein was only used in ELISA. As with humans, we found that there was concordance between ELISA and Chagas Sero *K*-SeT RDT, and that a greater number of *T. cruzi* seropositive samples tested by both methods were positive with the RDT, confirming the greater sensitivity.

Although we tested a limited number of dogs born after the community-wide insecticide spraying, dogs born prior to this intervention were over eight times more likely to be TSSApep-II/V/VI seropositive, showing the substantially higher risk before the spraying campaign, as well as cumulative risk with age. Qom dog ownership, rather than Creole, was also associated with higher prevalence of TSSApep-II/V/VI seropositivity (Table 2). Furthermore, dogs were more frequently born in Qom communities rather than Creole, which influenced the age that the dogs entered the household, increasing the likelihood of the dog being exposed to triatomines [25]. Owners were asked if the dogs were hunters or guardians and whether the dogs slept inside or outside the domicile; in both of these categories there were not statistically significant differences in Chagas Sero-*K* SeT result, however, in both cases the categorical divisions may not be entirely definitive.

*Trypanosoma cruzi* infections in cats are not uncommon, and also occur in domestic mice, which are caught and eaten by cats [46]; however, to our knowledge this is apparently the first application of *T. cruzi* lineage-specific serology to cats. TSSApep-II/V/VI positive cats had no association with the environmental and behavioural variables listed in Table 2 (data not shown). The Chagas Sero *K*-SeT failed with cats, not unexpectedly; the utility of Protein A, produced naturally by *Staphylococcus aureus*, rather than Protein G, for binding feline IgG has been reported [47, 48].

Both lineage-specific serology and genotyping indicated the predominance of TcII/V/VI in this endemic region of the Gran Chaco. The Chagas Sero *K*-SeT RDT demonstrated similar prevalence and clustering in humans and dogs, with ELISAs showing prominent TcV/VI infections in dogs and cats. Half of the dogs tested here that reacted by ELISA with TSSApep-II/V/VI also reacted with TSSApep-V/VI. TcV and TcVI are the most common genotypes infecting dogs and cats in this area [33].

Genotyping confirmed the association of TcIII with armadillos [49, 50]. As with the single TcIII infected dog, the Chagas Sero *K*-SeT positivities imply that both that dog and this armadillo were co-infected with TcII, TcV or TcVI. There is clearly a need for more extensive sampling among armadillos and sylvatic hosts generally. As with felines, the IgG-binding capacity of Protein A has been exploited in studies on armadilloes (*D. novemcinctus*), including the use of Protein A-sepharose columns to isolate Ig [51] and of HRP-conjugated Protein A in ELISA to recognise IgG [52].

There is as yet no reliably effective lineage-specific serology for TcI. Reasons for this are unclear, but may be due to the predicted low antigenicity of this isoform of TSSA [17] and perhaps associated with the lack of an ascribed function for TSSA-I, in contrast to TSSA-II/V/VI [53]. Thus, we cannot exclude some likely co-infections of TcI among the domestic and peridomestic transmission cycles at these study sites. There are relatively low sensitivity ELISAs for TcIII and TcIV [17, 36, 54], and more robust antigens for these lineages would greatly facilitate the study of ecological associations. However, for TcII/V/VI, we have proven here the practicality of deploying lineage-specific serology for surveillance and for enhancing understanding of transmission cycles, and the Chagas Sero *K*-SeT RDT, which is applicable in the field, can give a result in 15 minutes with minimal sample quantities (of whole blood, serum or plasma). Clearly, resolution of the molecular epidemiology of Chagas disease will also continue to benefit from further comparative genomics of *T. cruzi* isolates [55]. Nevertheless, the development of highly sensitive lineage-specific RDTs for all lineages, equally effective for both humans and a wide range of animals, with the aid of Protein G and Protein A detection, would be of great value. This would also allow the enigmatic issue of association of genetic lineage with pathology and prognosis of human Chagas disease to be re-addressed efficiently, and more widely [23].

We acknowledge that the samples used here represent single time-point sampling; however, they provide an antibody profile resultant from both historical and recent *T. cruzi* infections, although that profile may not be comprehensive.

## Conclusions

We have shown that lineage-specific serology can identify *T. cruzi* infecting lineage, without parasite isolation and genotyping. Furthermore, ELISA is replaceable by an at least equally sensitive RDT, the Chagas Sero *K*-SeT, which incorporates Protein G detection, and is thus directly applicable to humans and several other mammalian species. We assessed lineage distribution among 83% of the *T. cruzi*- seropositive human population, showing a statistically significant association of TSSApep-II/V/VI recognition with locality, and with increasing and decreasing age within the Qom and Creole populations, respectively. For dogs TSSApep-II/V/VI seroprevalence was linked to birth before the insecticide spraying programme and with Qom households. The Chagas Sero *K*-SeT is a low cost RDT, applicable for in-the-field surveillance, which can enhance understanding of the transmission pathways and clustering of the lineages, the epidemiology of Chagas disease and the risk of its further emergence from sylvatic cycles. Further research is required to produce corresponding lineage-specific RDTs for *T. cruzi* lineages TcI, TcIII and TcIV, particularly for TcI.

## Supplementary information

**Additional file 1: Table S1.** *Trypanosoma cruzi* lineage-specific peptides (TSSApep), with polymorphisms underlined. **Table S2.** Available corresponding *T. cruzi* genotyping information from humans and animals tested by TSSA lineage-specific serology.

## Data Availability

The dataset supporting the conclusions of this article are included within the article and its additional files.

## Abbreviations

ELISA: enzyme-linked immunosorbent assay
GLM: generalized linear model
IFAT: immunofluorescence antibody test
IgG: immunoglobulin G
PCR: polymerase chain reaction
TSSA: trypomastigote small surface antigen
TSSApep: lineage-specific TSSA peptide
RDT: rapid diagnostic test
