# Supplementary material for: Lineage-specific rapid diagnostic tests can resolve Trypanosoma cruzi TcII/V/VI ecological and epidemiological associations in the Argentine Chaco
Source: Parasit Vectors. 2019 Sep 16;12:424. doi: 10.1186/s13071-019-3681-7 (PMC6746045; doi:10.1186/s13071-019-3681-7)
Supplement: Supplementary file 1 — Additional file 1: Table S1. Trypanosoma cruzi lineage-specific peptides (TSSApep), with polymorphisms underlined. Table S2. Available corresponding T. cruzi genotyping information from humans and animals tested by TSSA lineage-specific serology. [file 13071_2019_3681_MOESM1_ESM.docx]

**Additional file 1: Table S1.** *Trypanosoma cruzi* lineage-specific peptides (TSSApep), with polymorphisms underlined.

| **Peptide** | **Amino acid sequence** |
| --- | --- |
| TSSApep-II/V/VI | GTENKPATGEAPSQPG |
| TSSApep-III | GTEKKAAAGEAPSPSG |
| TSSApep-IV | GTDKKTAAGEAPSPSG |
| TSSApep-V/VI | GTENKPAAGEAPSQPG |

**Additional file 1:** **Table S2.** Available corresponding *T. cruzi* genotyping information from humans and animals tested by TSSA lineage-specific serology. ND = not done.

| **Host** | **Sample** | **Genotype** | **Chagas Sero K- SeT** | **TSSApep ELISA** |
| --- | --- | --- | --- | --- |
| **Human** | 3_900 | TcV | Negative | ND |
|  | 3_870 | TcVI | Negative | ND |
|  | 3_1296 | TcV or TcV and TcVI | Positive | ND |
|  | 3_1195 | TcV or TcV and TcVI | Negative | ND |
|  | 3_1194 | TcV | Positive | ND |
|  | 3_1052 | TcV | Negative | ND |
|  | 3_1150 | TcV | Positive | ND |
|  | 3_1149 | TcV | Negative | ND |
|  | 3_1151 | TcV | Positive | ND |
|  | 3_994 | TcV | Positive | ND |
|  | 3_1039 | TcV or TcV and TcVI | Positive | ND |
|  | 3_1603 | TcV or TcV and TcVI | Negative | ND |
|  | 3_1697 | TcV | Negative | ND |
|  | 3_815 | TcV | Positive | ND |
|  | 3_1481 | TcV | Positive | ND |
|  | 3_816 | TcV | Positive | ND |
|  | 3_2093 | TcV | Positive | ND |
|  | 2_82 | TcVI | Positive | ND |
|  | 2_109 | TcV or TcV and TcVI | Positive | ND |
|  | 4_190 | TcV | Positive | ND |
|  | 4_191 | TcV | Positive | ND |
|  | 4_194 | TcV | Negative | ND |
|  | 4_205 | TcV | Positive | ND |
|  | 4_206 | TcV or TcV and TcVI | Positive | ND |
|  | 4_208 | TcV | Positive | ND |
|  | 2_211 | TcV | Positive | ND |
|  | 4_214 | TcV | Positive | ND |
|  | 2_228 | TcV | Positive | ND |
|  | 2_238 | TcV | Positive | ND |
|  | 4_243 | TcV | Positive | ND |
|  | 4_247 | TcV | Positive | ND |
|  | 4_249 | TcV | Positive | ND |
|  | 4_256 | TcV or TcV and TcVI | Positive | ND |
|  | 4_269 | TcV or TcV and TcVI | Positive | ND |
|  | 4_273 | TcV | Negative | ND |
|  | 2_414 | TcVI | Positive | ND |
|  | 2_643 | TcV | Negative | ND |
|  | 2_851 | TcV or TcV and TcVI | Positive | ND |
| **Dog** | 17 | TcVI | Positive | II/V/VI |
|  | 18 | TcVI | Positive | II/V/VI |
|  | 21 | TcVI | Positive | No TSSApep reaction |
|  | 63 | TcVI | Positive | No TSSApep reaction |
|  | 71 | TcVI | Positive | II/V/VI |
|  | 77 | TcVI | Positive | II/V/VI |
|  | 81 | TcIII | Positive | II/V/VI |
|  | 101 | TcVI | Positive | II/V/VI; V/VI |
|  | 103 | TcVI | Positive | No TSSApep reaction |
|  | 117 | TcVI | Positive | II/V/VI; V/VI |
|  | 152 | TcVI | Positive | II/V/VI; V/VI |
|  | 154 | TcVI | Positive | II/V/VI |
|  | 157 | TcVI | Positive | II/V/VI; V/VI |
|  | 272 | TcII/V/VI | Positive | No TSSApep reaction |
|  | 274 | TcII/V/VI | Negative | No TSSApep reaction |
|  | 276 | TcII/V/VI | Negative | No TSSApep reaction |
|  | 284 | TcVI | Positive | II/V/VI |
| **Cat** | 62 | TcVI | N/A | No TSSApep reaction |
|  | 78 | TcVI | N/A | No TSSApep reaction |
|  | 100 | TcVI | N/A | No TSSApep reaction |
|  | 132 | TcII/V/VI | N/A | No TSSApep reaction |
| **Armadillo** | 1 | TcIII | Negative | ND |
|  | 13 | TcIII | Negative | ND |
|  | 27 | TcIII | Positive | ND |
|  | 33 | TcIII | Negative | ND |
|  | 43 | TcIII | Negative | ND |
|  | 44 | TcIII | Negative | ND |
